# HMC05, Herbal Formula, Inhibits TNF-**α**-Induced Inflammatory Response in Human Umbilical Vein Endothelial Cells

**DOI:** 10.1093/ecam/nep126

**Published:** 2011-06-23

**Authors:** Jong Suk Lee, Su-Young Park, Dinesh Thapa, Ah Ra Kim, Heung-Mook Shin, Jung-Ae Kim

**Affiliations:** ^1^College of Pharmacy, Yeungnam University, Gyeongsan 712-749, Republic of Korea; ^2^Department of Physiology, College of Oriental Medicine, Dongguk University, Gyoungju 780-714, Republic of Korea

## Abstract

Vascular inflammation has been implicated in the progression of cardiovascular diseases such as atherosclerosis. In the present study, we found that HMC05, an extract from eight different herbal mixtures, dose-dependently inhibited tumor necrosis factor-**α** (TNF-**α**)-induced adhesion of monocytes to endothelial cells. Such inhibitory effect of HMC05 correlated with suppressed expression of monocyte chemoattractant protein-1, CC chemokine receptor 2, vascular cell adhesion molecule-1 and intercellular cell adhesion molecule-1. In addition, HMC05 significantly inhibited production of reactive oxygen species (ROS) and nuclear factor (NF)-**κ**B activation by TNF-**α**. Those inhibitory effects of HMC05 (1–10 **μ**g mL^−1^) on the TNF-**α**-induced inflammatory event was similar to those of berberine (1–10 **μ**M), which is a major component of HMC05 and one of herbal compounds known to have vasorelaxing and lipid-lowering activities. However, berberine significantly reduced the viability of HUVECs in a time- and concentration-dependent manner. In contrast, HMC05 (1–10 **μ**g ml^−1^) did not affect the cell viability for up to 48 h treatment. In conclusion, we propose that HMC05 may be a safe and potent herbal formula against vascular inflammation, and its action may be attributable to the inhibition of ROS- and NF-**κ**B-dependent expression of adhesion molecules and chemokines.

## 1. Introduction

Vascular endothelium plays a central role in modulating the inflammatory response in a way that they attract inflammatory cells, activate the coagulant and complement systems and increase vascular permeability [1]. Activation of endothelial cells by pro-inflammatory mediators express increased level of adhesion molecules such as vascular cell adhesion molecule-1 (VCAM-1), E-selectin and intercellular cell adhesion molecule-1 (ICAM-1) and chemokines, which facilitate adhesion of monocytes to endothelial cells [2, 3]. Such endothelial activation is one of the earliest and important processes in the pathophysiology of vascular diseases such as atherosclerosis and hypertension [4, 5]. Production of pro-inflammatory cytokines such as tumor necrosis factor-*α* (TNF-*α*) can be stimulated by various pathophysiological phenomena including modified low density lipoprotein (LDL) [6, 7], hemodynamic stress [8, 9] and hypertension [10].

HMC05 is a water extract of eight herbal drug mixtures originated and modified from a popular traditional herbal medicine, Banhabackchulchunmatang [11]. Previous studies on each of herbal drug components have revealed protective activities against atherosclerosis and inflammation through promotion of anti-inflammatory mediator production [12, 13], and inhibition of peroxynitrite-induced oxidative damage [14]. HMC05 has showed an anti-atherosclerotic activity through inhibition of pro-inflammatory cytokine production in a recent study with standardized HMC05 based on berberine and hesperidine [15]. However, the effects of HMC05 on endothelial inflammatory response have not been clearly demonstrated. In addition, previously, we have shown that berberine, an alkaloid isolated from the traditional Chinese herbal medicine, huanglian (*Coptis chinensis*), exert a cytotoxic effect on endothelial cells, whereas a berberine-containing herbal formula, Zoagumhwan, does not possess cytotoxicity on the cells [16]. HMC05, which contains berberine as a major component, has not been clearly demonstrated for its cytotoxicity profile on endothelial cells. Therefore, in the present study, we investigated the effects of HMC05 on TNF-*α*-induced inflammatory response and viability of endothelial cells in comparison to the effect of berberine alone.

## 2. Methods

### 2.1. Materials

Human umbilical vein endothelial cell line (HUVEC) and the human premonocytic cell line U937 were purchased from Clonetics (San Diego, CA, USA) and American Type Culture Collection (ATCC, VA, USA), respectively. Endothelial growth medium (EGM)-2 bullet kit which contains an endothelial cell basal medium (EBM)-2 and EGM-2 SingleQuots (supplements and growth factors) were purchased from Cambrex (San Diego). Fetal bovine serum (FBS) was obtained from Hyclone (Logan, UT, USA), and penicillin and streptomycin were from Gibco BRL (Rockville, MD, USA). 3-[4,5-dimethylthiazol-2-yl]-2,5-diphenyltetrazolium bromide (MTT), sodium pyruvate, berberine, and 2′,7′-dichlorodihydrofluorescein diacetate, acetyl ester (DCF-DA) were from Sigma-Aldrich (St Louis, MO, USA). Antibodies against NF-*κ*B (p65) and lamin B were purchased from Santa Cruz Biotechnology (Santa Cruz, CA, USA). Mouse monoclonal antibodies (mAbs) against human ICAM-1 and VCAM-1 were purchased from R&D Systems (Minneapolis, MN, USA), and rabbit polyclonal anti-CCR2 was from Abcam (Cambridge, UK).

### 2.2. Herbal Composition and Preparation of Water Extract (HMC05)

All the herbs used for HMC05, *Pinelliae ternate* Ten. Ex Breitenb., *Atractylodes macrocephala* Koidz., *Gastrodia elata* Blume, *Citrus unshiu* Marcow., *Poria cocos* Wolf, *Crataegus pinnatifida* Bunge var. *typica* C. K. Schneider, *Siegesbeckia pubescens* Makio. and *Coptidis japonica* Makino, were purchased from oriental drug stores (Kyungdong herb market, Seoul, Korea). The herbs were identified at the Oriental Medical College, Dongguk University (Gyoungju, Korea), where voucher specimen was preserved. The herbs had a moisture content of <10% by weight. HMC05 was extracted with 500 ml of distilled water under reflux for 3 h by boiling the formula, filtered with 10 *μ*m cartridge paper and concentrated to about 50 ml with a rotary evaporator at 50°C under vacuum and freeze-dried to dryness. It was standardized on the basis of berberine and hesperidine, using HPLC under acetonitrile: 0.1% formic acid gradient over a period of 25 min: acetonitrile 0–10%, 0–10 min; 10–40%, 10–20 min; 40–90%, 20–25 min [15]. The content of berberine and hesperidine in HMC05 was 1.93 ± 0.11% and 1.02 ± 0.11%, respectively.

### 2.3. Cell Culture

HUVECs were grown on 0.2% gelatin-coated flask with EBM-2 medium supplemented with 2% FBS, ascorbic acid, hydrocortisone, human fibroblast growth factor, vascular endothelial growth factor, human epidermal growth factor, long R insulin-like growth factor-1, gentamicin sulfate (GA-1000) and heparin. HUVECs between passage 2 and 6 were used in the experiments.

U937 cells were maintained in a RPMI 1640 medium supplemented with 10% FBS, 1 mM sodium pyruvate, 100 IU ml^−1^ penicillin and 100 *μ*g ml^−1^ of streptomycin. After attaining confluence, the cells were subcultured by splitting 1 : 5 ratio.

### 2.4. Measurement of Cell Viability

The cell viability was assessed using the MTT staining method. The cells from 4- to 5-day-old cultures were seeded in 96-well plates at a density of 1 × 10^4^ cells well^−1^. The volume of the medium in the wells was 100 *μ*l. In the control experiments, the cells were grown in the same media containing the drug-free vehicle. The cells were incubated with the drug for 48 h, and then with 10 *μ*l of MTT (5 g l^−1^) for 4 h. Two hundred microliters of dimethyl sulfoxide were added to each culture and mixed by pipetting to dissolve the reduced MTT crystals. The relative cell viability was determined by measuring optical density in a microplate reader (Molecular Devices, Versa MAX Sunnyvale, CA, USA) at 540 nm.

### 2.5. Fluorescent Labeling of U937 Cells and Cell Adhesion Assay

U937 human premonocytic cells were used to investigate leukocyte-endothelial cell interaction [17], and these cells are known to express CCR2, a receptor for MCP-1 [18]. U937 cells were labeled with 2′,7′-*bis*(2-carboxyethyl)-5(6)-carboxyfluorescein acethoxymethyl ester (BCECF/AM, 10 *μ*g ml^−1^) for 1 h at 37°C. HUVECs cultured in 24-well plate were pretreated with HMC05 water extract or berberine for 30 min and then incubated with TNF-*α* for an additional 3 h. Then, HUVECs were co-incubated with BCECF/AM-prelabeled U937 cells (1 × 10^6^ cells well^−1^) for 30 min at 37°C. Non-adhering U937 cells were removed, and the cells were washed twice with PBS. A set of cells was taken and imaged by inverted microscopy connected to digital camera (TMS; Nikon, Japan), and in other sets, cells were lysed in 0.1% Triton X-100 in 0.1 mol l^−1^ Tris. Fluorescence was measured by using a Fluostar optima microplate reader (BMG LABTECH GmbH, Germany) using excitation at 485 nm and emission at 520 nm.

### 2.6. Quantitative Real-Time Reverse Transcription Polymerase Chain Reaction

Cells were collected and total RNA was extracted with a spin column using RNeasy kit (Qiagen, Hilden, Germany) according to the manufacturer's instructions. Isolated mRNA was reverse transcribed by using the Reverse Transcription kit (Qiagen). The gene expression levels were analyzed by quantitative real-time PCR system (Rotor-Gene 6000; Corbett, Sydney, Australia). Real-time PCR was performed using a SYBR Green PCR kit (Qiagen). The primer sequences for MCP-1, CCR2, VCAM-1 and ICAM-1 were as follows: MCP-1, sense 5′-TCGCGAGCTATAGAAGAATC-3′ and antisense 5′-AATCCTGAACCCACTTCT-3′; CCR2, sense 5′-CTGGTGTTCATCTTTGGTTT-3′ and antisense 5′-CCCACAATGGGAGAGTAATA-3′; VCAM-1, sense 5′-GGGAGCTCTGTCACTGTAAG-3′ and antisense 5′-ATCCGTATCCTCCAAAAACT-3′; ICAM-1, sense 5′-CCTTCCTCACCGTGTACTGG-3′ and antisense 5′-AGCGTAGGGTAAGGTTCTTGC-3′. The reaction mixture consisted of 2 *μ*l of cDNA template, 10 *μ*l of SYBR Green PCR master mix and 5 pmol of primers in total volume of 20 *μ*l. The cDNA was denatured at 95°C for 15 min followed by 40 cycles of PCR (95°C for 5 s, 55°C for 10 s, 72°C for 20 s). The mRNA levels of all genes were normalized using glyseraldehyde-3-phosphate dehydrogenase GAPDH (Qiagen) as an internal control.

### 2.7. Immunocytochemical Analysis for ICAM-1 and VCAM-1 Expression

The cellular localization of adhesion molecules, ICAM-1 and VCAM-1, were examined by an immunocytochemical method. HUVECs were cultured in sterile eight-well chambered slides (Nalge Nunc International, Naperville, IL, USA) with medium alone or medium containing HMC05 or vehicle for 1 h before an additional 3 h treatment of TNF-*α* (10 ng ml^−1^). The cells were then fixed with ice-cold methanol for 5 min at −20°C. After air drying for 10 min, the slides were blocked with 3% bovine serum albumin (BSA) for 1 h and incubated with ICAM-1 or VCAM-1 monoclonal antibody (1 : 20 dilution of a 200  *μ*g ml^−1^ solution) at 4°C overnight in humidified chamber. The cells were washed three times with TBS-T (20 mM Tris–HCl, pH 7.5, 500 mM NaCl, 0.1% Tween 20) for 5 min and then incubated with Alexa Fluor 488 donkey anti-mouse IgG (1 : 200) in TBS with 3% BSA for 1 h at room temperature. After washing in TBS-T for 30 min, the cells were stained with 600 nM 4′,6-diamidino-2-phenylindole (DAPI) for 2 min. After several brief washings, the cells were mounted with Prolong Gold Antifade reagent and covered with a coverslip. Finally, the cells were observed using a Nikon microscope (TE-2000U) with appropriate excitation/emission filter pairs.

### 2.8. Measurement of Intracellular Reactive Oxygen Species

Intracellular reactive oxygen species (ROS) generation was measured using 2′,7′-dichlorofluorescein diacetate (DCF-DA; Sigma), a fluorescent dye. Confluent cells were subjected to serum starvation (0.5% FBS) for 24 h and then used for experiments. Starved cells were treated with HMC05 or berberine in the presence of TNF-*α* (10 ng ml^−1^) for 1 h. The cells were loaded with DCF-DA (5 *μ*M) for 5 min at 37°C, and then, imaged by inverted fluorescence microscopy (TE2000-U; Nikon, Japan), and analyzed with an image analysis system (ImageInside Ver 2.32).

### 2.9. Protein Extraction and Western Blot Analysis

Cells were incubated with TNF-*α* in the presence or absence of HMC05. For the detection of NF-*κ*B in HUVECs, nuclear protein extraction was carried out by using an NE-PER kit as per manufacturer's instructions (Pierce, Rockford, IL, USA). In case of CCR2 detection, total proteins were extracted from U937 cells using RIPA buffer containing protease inhibitor cocktail (Sigma). Protein content was measured with BCA protein assay reagent (Pierce). Equal amount of proteins (20 *μ*g nuclear or 70 *μ*g total proteins) were separated on 10% SDS–PAGE and transferred to nitrocellulose membranes. The membranes were blocked with 5% nonfat milk in TBS-T for 2 h, and incubated with specific antibodies in TBS containing 1% non-fat milk at 4°C overnight. After washing three times with TBS-T, the membranes were hybridized with horseradish peroxidase-conjugated secondary antibody in skim-milk-TBS for 1 h at room temperature. The immunoreactive proteins were visualized using an ECL kit.

### 2.10. NF-*κ*B Reporter Gene Dual-Luciferase Assay

NF-*κ*B transactivation was studied by using NF-*κ*B luciferase reporter construct (firefly luciferase) in conjunction with 0.05 *μ*g/ml of pRL-TK (renilla luciferase as a transfection control) using lipofectamine transfection reagent (Invitrogen, CA, USA) according to the manufacturer's instructions. HUVECs (1 × 10^5^ cells well^−1^) were incubated with transfection mixture at 37°C for 5 h, mixed with the same volume of growth medium, and kept in an incubator at 37°C overnight. These cells were then treated with HMC05 or BER in the presence of 10 ng ml^−1^ TNF-*α*. After 3 h, the cells were washed with PBS and then lysed by repeated freezing and thawing. Cells were then scraped gently and the lysates were centrifuged at 10 000 rpm for 5 min. Firefly and renilla luciferase activities were measured using Dual-Luciferase Reporter Assay Kit (Promega Corporation, Madison, WI, USA) on a Turner TD20/20 luminometer (Turner Biosystems, CA, USA).

### 2.11. Statistical Analysis

The data are expressed as mean ± standard error of mean (SEM) of more than three independent experiments and were analyzed using one-way analysis of variance (ANOVA) and the Student—Newman—Keul's test for individual comparisons. *P* values of < .05 were considered statistically significant.

## 3. Results

### 3.1. HMC05 Inhibits Monocyte Adhesion to Endothelial Cells Induced by oxLDL and TNF-*α*

**Figure 1:**
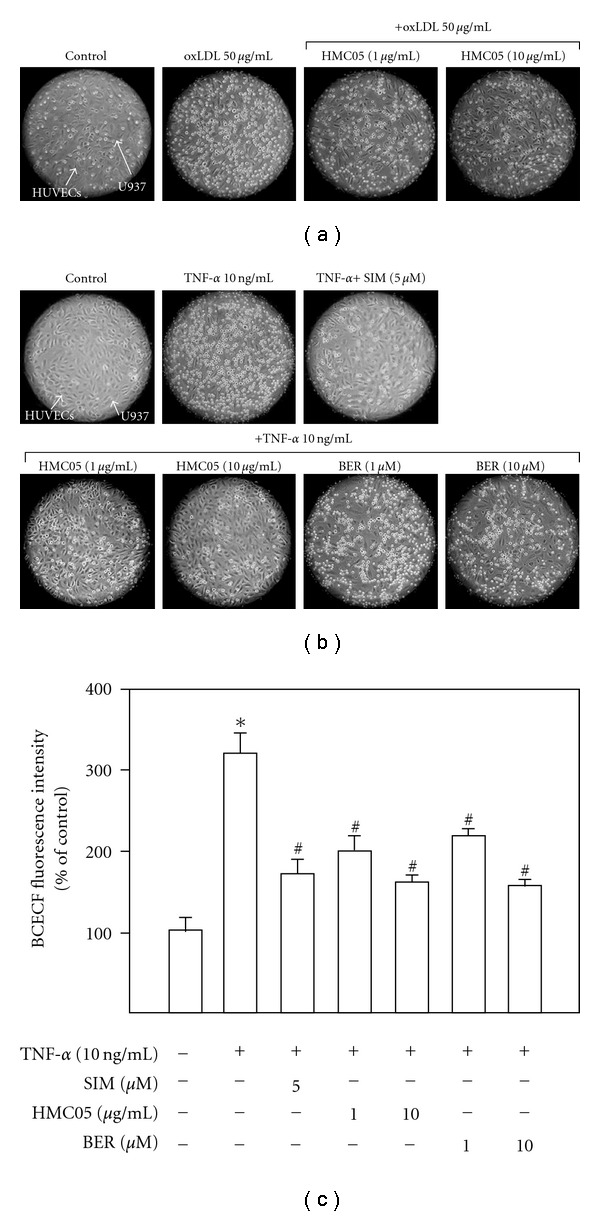
Inhibitory effects of HMC05 and berberine on oxLDL- and TNF-*α*-induced monocyte adhesion to endothelial cells. The adhesion of fluorescence-labeled U937 cells to HUVECs was detected by microscopic images ((a) and (b)), and quantitated by using a fluorescence-detecting microplate reader (c). HUVECs were pretreated with different concentrations of HMC05 (1 and 10 *μ*g ml^−1^), simvastatin (5 *μ*M) or berberine (1 and 10 *μ*M) for 30 min before oxLDL (50 *μ*g ml^−1^) and TNF-*α* (10 ng ml^−1^) treatment for 3 h. Data are expressed as the mean ± SEM of three independent experiments with duplicates. *P < .01 compared with untreated control group. ^#^ *P* < .01 compared with TNF-*α*-treated group.

We first examined the effect of HMC05 on the oxidized LDL (oxLDL)-induced vascular inflammation by measuring the adhesion of inflammatory leukocytes to endothelial cells, a crucial step in inflammatory process. As shown in Figure 1(a), HMC05 inhibited oxLDL-induced adhesion of leukocytes to HUVECs. Since in a variety of vascular diseases including atherosclerosis, TNF-*α* level is increased in the lesion site and contributes to vascular inflammatory process [19, 20], we then examined the effect of HMC05 on TNF-*α*-treated endothelial response. HUVECs stimulated with TNF-*α* (10 ng ml^−1^) for 3 h significantly increased adhesion of leukocytes to the endothelial cells compared with untreated (control) cells (Figures 1(b) and 1(c)). The TNF-*α*-induced adhesion was significantly inhibited by HMC05 (1 and 10 *μ*g ml^−1^) in a concentration-dependent manner. The inhibitory activity of HMC05 (1–10 *μ*g ml^−1^) on TNF-*α*-induced adhesion of monocytes to HUVECs was similar to those of berberine (1–10 *μ*M), a major component of HMC05, or simvastatin (5 *μ*M), a hypolipidemic and cardiovascular risk-reducing drug.

### 3.2. Inhibitory Effects of HMC05 on TNF-*α*-Induced Expression of Chemokine, MCP-1 and Adhesion Molecules, VCAM-1 and ICAM-1, in HUVECs

**Figure 2:**
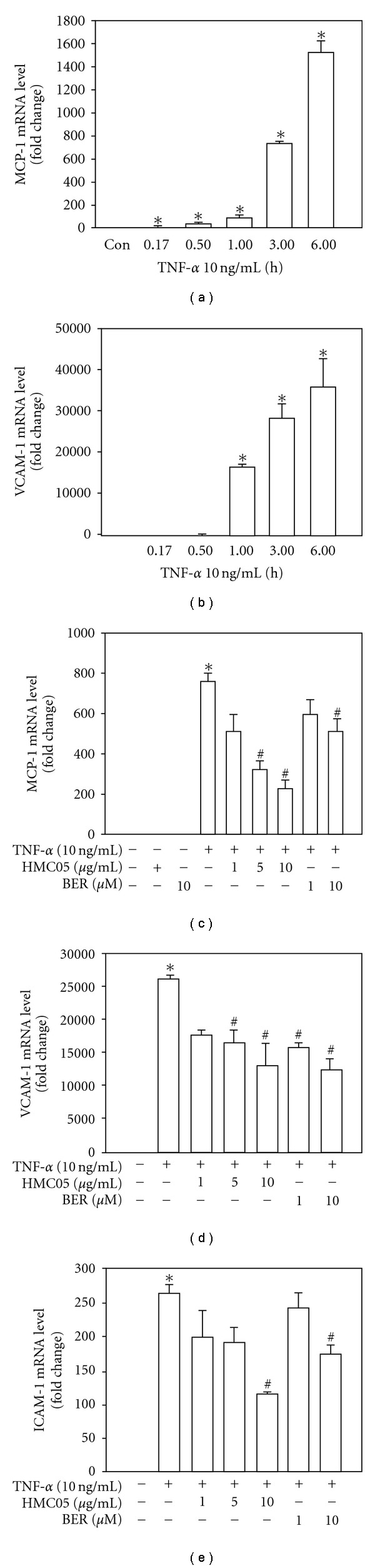
Inhibitory effects of HMC05 and berberine on TNF-*α*-induced mRNA expression of MCP-1, VCAM-1 and ICAM-1 in HUVECs. Serum-starved HUVECs were treated with HMC05 and berberine in the absence ((a) and (b)) or presence (c–e) of TNF-*α* (10 ng ml^−1^). After 3 h, the cells were harvested, and the mRNA expression of MCP-1 ((a) and (c)), VCAM-1 ((b) and (d)) and ICAM-1 (e) was quantified by real-time RT-PCR. Data are expressed as the mean ± SEM of four independent experiments with duplicates. *P < .05 compared with untreated control group. ^#^ *P* < .05 compared with TNF-*α*-treated group.

**Figure 3:**
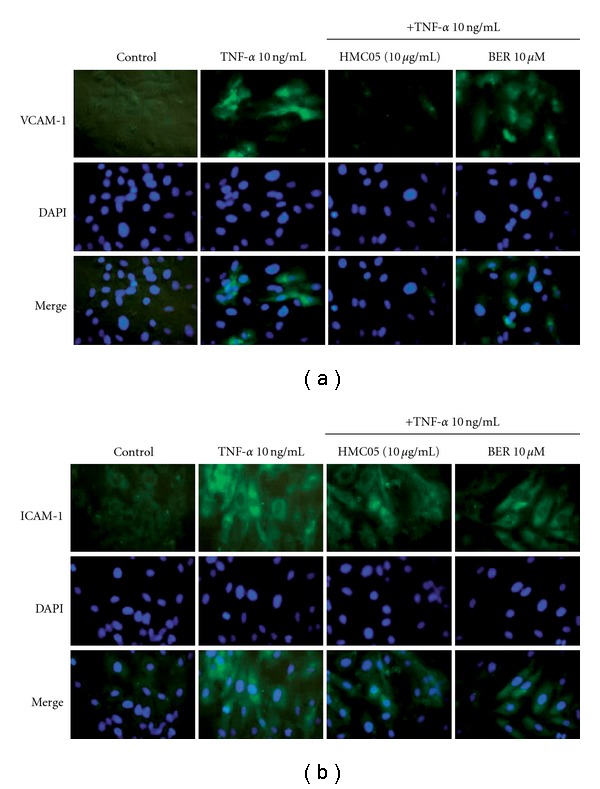
Inhibitory effects of HMC05 and berberine on TNF-*α*-induced protein expression of adhesion molecules, VCAM-1 (a) and ICAM-1 (b), in HUVECs. Serum-starved HUVECs were treated with HMC05 and berberine in the absence or presence of TNF-*α* (10 ng ml^−1^) for 3 h. After fixation with methanol, immunocytochemical analysis was performed as described in ‘Materials and methods' section. The result shown is a representative of three independent experiments.

The increased expression of chemokines and cell adhesion molecules on endothelial cells promotes the adhesion of leukocytes, which is regarded as the molecular basis for the inflammatory response observed in various diseases [20–22]. Real-time reverse transcription polymerase chain reaction (RT–PCR) was carried out to examine the effects of HMC05 on the mRNA expression of MCP-1, one of the key factors to initiate inflammatory process in vascular inflammation, and adhesion molecules, VCAM-1 and ICAM-1. Treatment of HUVECs with TNF-*α* increased MCP-1 (Figure 2(a)) and VCAM-1 (Figure 2(b)) mRNA level in a time-dependent manner. Treatment with HMC05 concentration-dependently suppressed the TNF-*α*-induced mRNA expression of MCP-1 (Figure 2(c)), VCAM-1 (Figure 2(d)) and ICAM-1 (Figure 2(e)). Similarly, berberine treatment also inhibited the TNF-*α*-induced MCP-1, VCAM-1 and ICAM-1 mRNA expressions. The degree of reduction in VCAM-1 expression by HMC05 (10 *μ*g ml^−1^) was similar to the effect of berberine (10 *μ*M), whereas suppression of MCP-1 and ICAM-1 expression by HMC05 was stronger than by berberine. To further assess the effect of HMC05 on protein expression of the adhesion molecules, immunocytochemistry were performed. As shown in Figures 3(a) and 3(b), VCAM-1 and ICAM-1 protein expression levels were high in TNF-*α*-stimulated HUVECs, which was suppressed by HMC05 and berberine treatment.

### 3.3. CCR2 Expression in TNF-*α*-treated Human Monocytic U937 Cells Was Inhibited by HMC05

**Figure 4:**
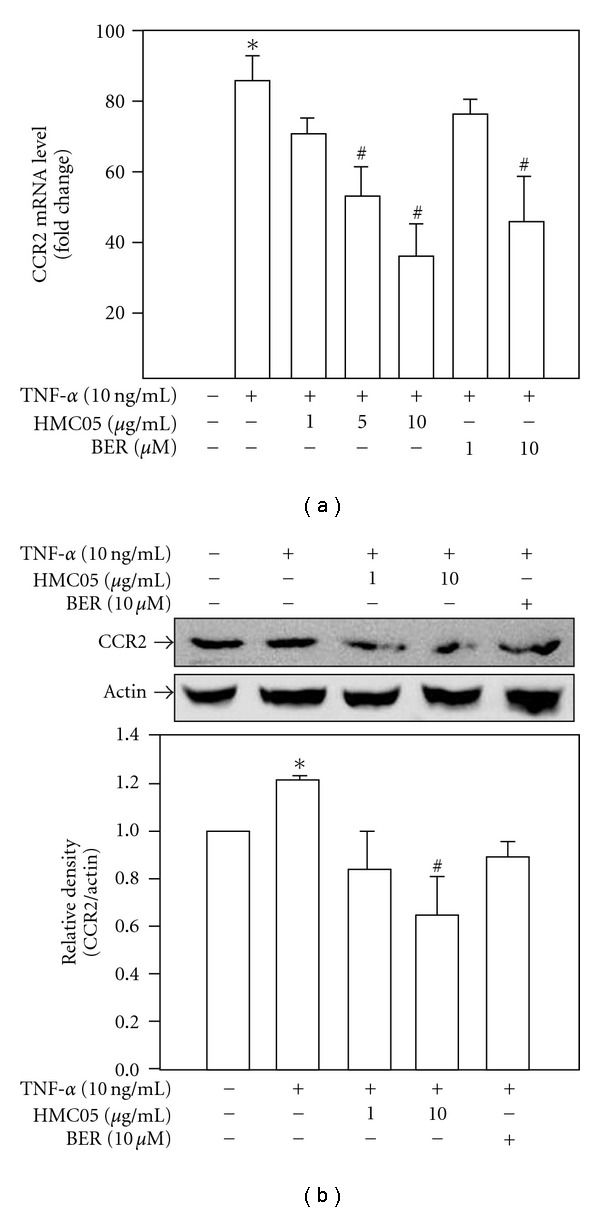
Inhibitory effects of HMC05 and berberine on TNF-*α*-induced CCR2 mRNA (a) and protein expression (b) in U937 cells. Serum-starved cells were treated with HMC05 and berberine in the presence of TNF-*α* for 3 h. CCR2 mRNA level was examined by real-time RT–PCR (a), and the data are expressed as the mean ± SEM of four independent experiments with duplicates. CCR2 protein expression was examined by western blot analysis (b). The blot is a representative of three independent experiments, and the bar graphs represent relative density of CCR2/Actin. Data are expressed as the mean ± SEM of three independent experiments. *P < .01 compared with untreated control group. ^#^ *P* < .01 compared with TNF-*α*-treated group.

Since MCP-1 binds only to CCR2 [23], the expression of CCR2 plays an important role in monocyte recruitment and in many inflammatory states of vessels. The CCR2 mRNA expression was increased by the treatment with TNF-*α* for 3 h in U937 cells, but this effect was significantly inhibited by co-treatment with HMC05 (10 *μ*g ml^−1^) (Figure 4(a)). The effect of berberine (10 *μ*M) on the TNF-*α*-induced CCR2 expression was similar to that of HMC05. Similarly, we also noted the protein expression of CCR2 in U937 cells was increased by TNF-*α* in a western blot analysis, which upon HMC05 treatment, significantly downregulated in a concentration-dependent manner (Figure 4(b)).

### 3.4. Geranylgeranyl Pyrophosphate Reverses the Inhibitory Activity of HMC05 on TNF-*α*-Induced ROS Increase

**Figure 5:**
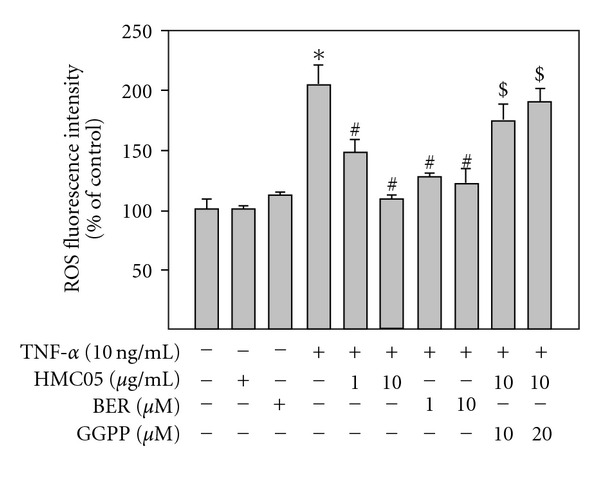
HMC05 suppresses TNF-*α*-induced geranylgeranyl isoprenoid-dependent ROS generation. HUVECs were treated with TNF-*α* in the presence or absence of HMC05 and/or GGPP for 1 h, and incubated with DCF-DA (5 *μ*M) for an additional 5 min. The intensity of DCF fluorescence detecting intracellular ROS was analyzed by using ImageInside program. Data are expressed as the mean ± SEM of three independent experiments. *P < .01 compared with untreated control group. ^#^ *P* < .01 compared with the TNF-*α*-treated group. ^$^ *P* < .01 compared with TNF-*α* and HMC05-treated group.

Since TNF-*α* increases ROS production which is a major cause of vascular inflammation, we examined the effect of HMC05 on TNF-*α*-induced ROS increase by measuring DCF fluorescence using a microfluorometry. Co-treatment of the cells with HMC05 and TNF-*α* significantly inhibited the TNF-*α*-induced ROS production (Figure 5). Similarly, treatment with berberine also suppressed the TNF-*α*-induced ROS increase. It is well known that TNF-*α*-induced ROS increase in endothelial cells is associated with activation of NADPH oxidase through prenylation of Rac1 by geranylgeranyl pyrophosphate (GGPP) [24–26]. To assess the involvement of NADPH oxidase through Rac prenylation in TNF-*α*-induced ROS production, we examined the effects of exogenous application of GGPP. The pretreatment with exogenously applied GGPP in combination with HMC05 completely reversed the inhibitory effects of HMC05 on TNF-*α*-induced ROS generation (Figure 5).

### 3.5. Suppression of NF-*κ*B Transcriptional Activity by HMC05 in HUVECs Treated with TNF-*α*

**Figure 6:**
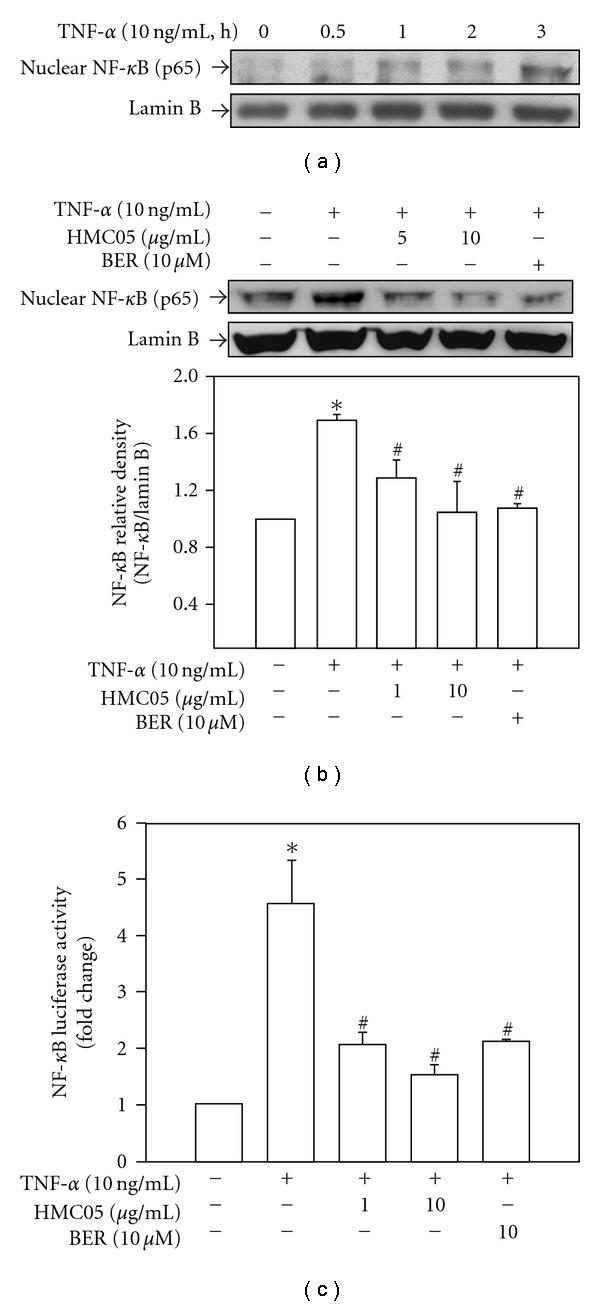
Inhibitory effects of HMC05 and berberine on TNF-*α*-induced NF-*κ*B activation in HUVECs. The cells were incubated with TNF-*α* alone for the designated time (a), or in the presence of HMC05 and berberine for 3 h (b). The nuclear extracts (20 *μ*g protein) were separated on SDS–PAGE, transferred to a nitrocellulose membrane, and blotted with the anti-NF-*κ*B p65 subunit antibody. The blots are a representative of three independent experiments, and the bar graphs represent relative density of NF-*κ*B/Lamin B. (c) After HUVECs were transfected with NF-*κ*B luciferase (firefly) construct together with pRL-TK (renilla luciferase), the cells were stimulated with TNF-*α* in the presence or absence of HMC05 or berberine. Luciferase activities were determined using a dual-luciferase reporter assay kit and normalized to those of control values. The data shown are mean ± SEM of three independent experiments. *P < .05 compared with untreated control group. ^#^ *P* < .05 compared with the TNF-*α*-treated group.

NF-*κ*B is a well-known redox-sensitive transcription factor of various proinflammatory genes including cell adhesion molecules [22]. TNF-*α*-induced nuclear NF-*κ*B p65 accumulation (Figure 6(a)) was significantly suppressed by HMC05 and berberine (Figure 6(b)). Furthermore, in the cells transfected with NF-*κ*B-Luc plasmid (Figure 6(c)), TNF-*α*-stimulated increase in NF-*κ*B transcriptional activity was inhibited by HMC05 in a concentration-dependent manner. Such effect of HMC05 (1 *μ*g ml^−1^) was similar to that of berberine (10 *μ*M).

### 3.6. Differential Effects of HMC05 and Berberine on the Viability of HUVECs

**Figure 7:**
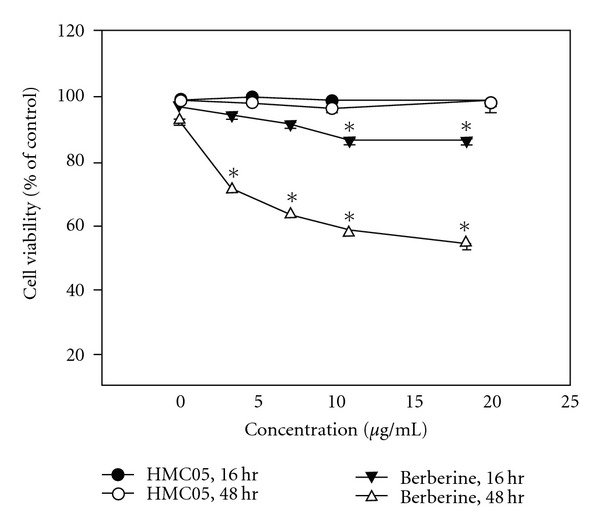
Effects of HMC05 and berberine on the viability of HUVECs. Cells were treated with different concentration of HMC05 and berberine for the indicated time, and then, cell viability was measured by MTT assay. The data are shown as mean ± SEM of three independent experiments with triplicates. *P < .01 compared with untreated control group.

To examine whether treatment of the cells with HMC05 affects viability of endothelial cells, HUVECs were treated with HMC05 or berberine in a different time- and concentration-scale. As shown in Figure 7, HMC05 treatment had no adverse effect on the viability of HUVECs (>95% cell viability). In contrast, berberine significantly reduced the viability of HUVECs in a time- and concentration-dependent manner.

## 4. Discussion

Pro-inflammatory cytokines are thought to be central players in the development of vascular inflammation, which leads to the development of cardiovascular complications [27, 28]. TNF-*α*, a commonly found cytokine in atherosclerotic lesions, induces cell adhesion molecules, which contribute to the inflammatory process [20]. In addition, it has been highlighted that cytokine-activated endothelial cells secrete monocyte-specific chemoattractant molecules to recruit monocytes at the sites of vascular injury and inflammation [29].

The present study clearly showed that HMC05 significantly inhibited oxLDL-and TNF-*α*-induced monocyte adhesion onto endothelial cells in along with reduced expressions of MCP-1, VCAM-1 and ICAM-1 in HUVECs. In addition, HMC05 also blocked the TNF-*α*-increased expression of CCR2, the MCP-1 receptor, in monocytes. Since the MCP-1/CCR2 binding is the major regulator of monocyte recruitment and seems to play a primary role in many inflammatory states of vessels, our results suggest that HMC05 is an effective formula for vascular inflammation by suppressing both the expression of endothelial chemokine and monocyte chemokine receptors.

Oxidative stress is a characteristic feature of the vascular inflammatory response and in the pathogenesis of atherosclerosis [30]. The expression of adhesion molecules on the endothelial cell surface during primary inflammatory response is dependent on the ROS-sensitive nuclear transcription factor NF-*κ*B and AP-1 activation [2, 31–33]. Our results showing that HMC05 inhibited TNF-*α*-induced ROS and NF-*κ*B translocation indicate that inhibitory effect of HMC05 on TNF-*α*-induced monocyte adhesion to endothelial cells is possibly mediated through suppression of ROS and subsequent NF-*κ*B activation.

Most importantly, our results clearly showed the difference between HMC05 and berberine, a major component of HMC05. The inhibitory effects of HMC05 on the monocyte-endothelial adhesion were similar to those of berberine, whereas HMC05 did not affect the cell viability in contrast to berberine. Although berberine is known to have lipid-lowering effect, and vasorelaxing activities as well as ameliorating the cardiovascular diseases such as hypertension and atherosclerosis [34–36], recent study revealed that berberine caused prothrombotic effects on endothelial cells, indicating the limitation on therapeutic application of berberine as a monotherapy [37]. Furthermore, the cytotoxic property of berberine is the most important and frequently tested biological activity [38]. Similar to the previous reports, in the present study, berberine showed significant cytotoxicity in HUVECs. However, HMC05 did not show any cytotoxic effect on HUVECs, suggesting that berberine-containing HMC05 may be a safer remedy in the treatment of vascular inflammation than berberine alone. Although we had not determined the amount and activity of exact component, HMC05 contains high levels of phenolic compounds that have wide range of biological activities including antioxidant effects. Recently, the main components of HMC05 were screened and identified as one flavonoid, hesperidin and three alkaloids, coptisine, palmatine and berberine by simultaneous determination method [39]. For example, hesperidin, one of the major components of HMC05, is also reported to have many biological effects including anti-inflammatory, antimicrobial, anticarcinogenic, antioxidant and radioprotective effects [40, 41]. Based on those reports, the reduced cytotoxicity of HMC05 compared with berberine alone may possibly be due to the compensating action of flavonoid, hesperidin, against cytotoxic nature of berberine.

**Figure 8:**
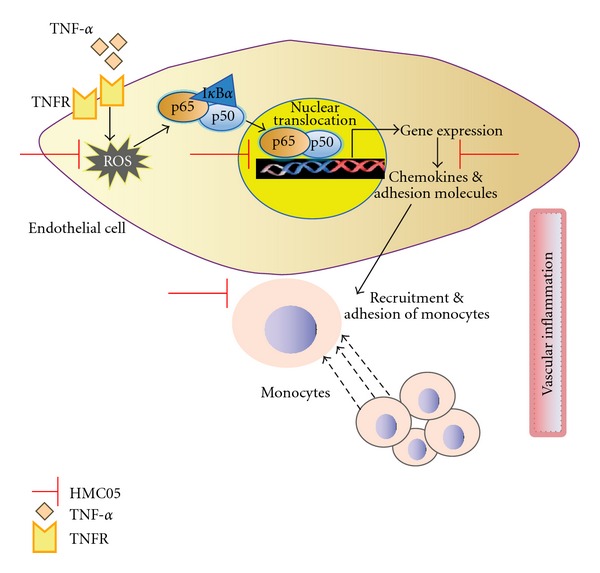
Schematic diagram showing signaling pathway HMC05 action against TNF-*α*-induced vascular inflammation. HMC05 may protect vascular inflammation through suppression of ROS production and the expression of NF-*κ*B-related adhesion molecules in endothelial cells, which in turn, prevents monocyte adhesion to endothelial cells. TNFR: tumor necrosis factor-*α* receptor. The solid arrow and broken arrow represent activation, and migration, respectively.

In conclusion, our results indicate that HMC05 may protect vascular inflammation through suppression of ROS production and regulation of the expression of NF-*κ*B-related adhesion molecules in endothelial cells (Figure 8).

## Funding

Korea Health 21 R&D Project, Ministry of Health & Welfare, Republic of Korea (grant no B080031) and the Regional Technology Innovation Program of the Ministry of Commerce, Industry and Energy (MOCIE) (grant no RTI04-01-04).
